# Sevoflurane protects rat brain endothelial barrier structure and function after hypoxia-reoxygenation injury

**DOI:** 10.1371/journal.pone.0184973

**Published:** 2017-10-12

**Authors:** Tanja Restin, Marie-Elisabeth Kajdi, Martin Schläpfer, Birgit Roth Z’graggen, Christa Booy, Claudia Dumrese, Beatrice Beck-Schimmer

**Affiliations:** 1 Institute of Anesthesiology, University Hospital Zurich, Zurich, Switzerland; 2 Institute of Physiology, Zurich Centre for Integrative Human Physiology, University of Zurich, Zurich, Switzerland; 3 Flow Cytometry Facility, University of Zurich, Zurich, Switzerland; 4 Department of Anesthesiology, University of Illinois at Chicago, Chicago, United States of America; Hungarian Academy of Sciences, HUNGARY

## Abstract

**Background:**

After cerebral injury blood-brain barrier disruption significantly impairs brain homeostasis. Volatile anesthetics have been shown to be protective in ischemia-reperfusion injury scenarios. Their impact on brain endothelial cells after hypoxia-reoxygenation (H/R) has not yet been studied in detail.

**Methods:**

Rat brain endothelial cells (RBE4) were exposed to severe hypoxia and reoxygenated in air in the presence or absence of sevoflurane. Changes in dextran permeability and architecture of the cellular junctional proteins ZO-1 and β-catenin were measured. To determine necrosis and apoptosis rate DNA content, LDH release and caspase activity were quantified. The role of vascular endothelial growth factor (VEGF) as an inflammatory mediator increasing vascular permeability was assessed. At the same time, it was evaluated if sevoflurane effects are mediated through VEGF. Results were analyzed by unpaired t-tests or one way-analysis of variance followed by Bonferroni’s correction.

**Results:**

H/R led to a 172% increase in permeability (p<0.001), cell swelling and qualitatively but not quantitatively modified expression of ZO-1, β-catenin and F-actin. In the presence of sevoflurane during reoxygenation, barrier function improved by 96% (p = 0.042) in parallel to a decrease of the cell size and less re-arranged junction proteins and F-actin. Sevoflurane-induced improvement of the barrier function could not be explained on the level of necrosis or apoptosis as they remained unchanged independent of the presence or absence of the volatile anesthetic. Increased expression of VEGF after H/R was attenuated by sevoflurane by 34% (p = 0.004). Barrier protection provided by sevoflurane was similar to the application of a blocking VEGF-antibody. Furthermore, the protective effect of sevoflurane was abolished in the presence of recombinant VEGF.

**Conclusions:**

In H/R-induced rat brain endothelial cell injury sevoflurane maintains endothelial barrier function through downregulation of VEGF, which is a key player not only in mediating injury, but also with regard to the protective effect of sevoflurane.

## Introduction

The blood-brain barrier (BBB) strictly controls and regulates molecular exchange between the cerebral and vascular compartments, thereby safeguarding cerebral function [1]. Large protein complexes of endothelial cells, which form tight and adherens junctions, limit the paracellular flux [2]. The tight junction protein zonula occludens (ZO-1) and the adherens junction protein β-catenin are thereby key components contributing to normal barrier function [3]. Experimental models suggest that the generation of brain edema after BBB breakdown involves an early phase of leakage after 4 hours [4]. Particularly in brain injury, this early phase of brain edema formation involves an increase of vascular endothelial growth factor (VEGF) [5], one of the most potent permeability factors [6] [7]. VEGF directly modifies endothelial cell junctions [8, 9] [10] as well as the actin cytoskeleton [11] and may therefore promote further disruption of the blood-brain barrier, finally leading to neuronal malfunction [12].

Volatile anesthetics such as sevoflurane have been shown to sustain tissue integrity after ischemia-reperfusion (hypoxia-reoxygenation, H/R) injury in the heart [13], the liver [14], the lung [15] and the kidney [16]. They also affect the BBB and its function, with some authors postulating neuroprotective effects of volatile anesthetics [17, 18]. So far, limited information is available regarding the interaction of these anesthetics with the endothelial component of the BBB.

Working from the hypothesis that sevoflurane improves H/R-induced endothelial barrier dysfunction, we investigated whether the application of sevoflurane alters the permeability of rat brain endothelial cell (RBE4) layers and modifies important tight and adherens junctions. VEGF as one of the key permeability factors was determined in order to gain more insight into the possible signaling pathway.

## Methods

### Cell culture

RBE4 rat brain endothelial cells (P. Couraud, Cochin Institute, University Descartes, Paris) were grown on collagen-coated plates (rat tail collagen, 30–100μg (cm^2^)^-1^ Sigma-Aldrich Chemie GmbH, Buchs, Switzerland) [19]. Media contained F10 and α-MEM in equal amounts, enriched with 10% fetal bovine serum (Life technologies, Zug, Switzerland), 2% 4-2- hydroxyethyl-1 piperazinethanesulfonic acid (Hepes) 1M (Sigma), 1ng/ml human basic fibroblast growth factor (PreproTech, London, UK) and 300μg ml^-1^ geneticin (Sigma). Cells were incubated in humidified room air (humidity 70–90%) enriched with 5% CO_2_.

### Hypoxia and reoxygenation injury with or without sevoflurane

For all experiments, cells were seeded at a density of 50,000 cells ml^-1^ and grown for 2–3 days to a confluency of 80–100%. Experiments started with either severe hypoxic (0.2% oxygen) or normoxic (21% oxygen) treatment for 24 hours. For hypoxic exposure, cells were transferred to an anaerobic work station (Concept 400M, Ruskinn Technology, Baker Company, Sanford, Maine USA), while control cells remained in the normal incubator. Reoxygenation with sevoflurane was performed in Oxoid chambers (Oxoid, Hampshire, UK) for 4 hours in a mixture of room air enriched with 2.2% sevoflurane (Baxter Schweiz AG, Volketswil, Switzerland) and 5% CO_2_, defined as postconditioning, in line with previous work carried out by our group [20].

### Permeability assays

For permeability analysis, RBE4 cells were grown on collagen-coated Transwell^TM^ chambers with 6.5mm-diameter polycarbonate inlays of pore size 0.4μm (Corning Incorporated, Corning, NY, USA). After exposure to H/R (or normoxia as control), medium was removed from the top compartment and fluorescein isothiocyanate (FITC)-dextran with a molecular weight of 40kD (Sigma) was added at a concentration of 1mg ml^-1^. FITC-dextran permeation to the lower compartment was measured after 10, 20 and 30 minutes for the clearance curve analysis and after 30 minutes for determination of permeability. The amount of FITC permeation was quantified with the aid of a fluorometer (Infinite 200 pro, Tecan, Männedorf, Switzerland) at an excitation wave length of 490nm and an emission wave length of 525nm. The cleared volume was plotted against time and the permeability coefficient P_e_ was calculated according to Rist et al. [21]: 1/PS = 1/me-1/mf and P_e_ = PS/S, where PS is defined as permeability surface area product, and S as surface area of the filter (0.331cm^2^). me and mf represent the slopes of the clearance curves of filters with (me) and filters without endothelial cells (mf) in ml = (cm^3^)min^-1^.

### Protein extraction

Cells were washed with ice-cold, phosphate-buffered saline (PBS, Kantonsapotheke Zurich, Switzerland; **S1 Table**) and homogenized in protein lysis buffer (containing 250mM Sucrose, 20mM Hepes, 10mM KCl, 1.5mM MgCl_2_, 1mM EDTA, 1mM EGTA, 1mM DTT). 100μl of protease inhibitor mix (8340) and 50μl phosphatase inhibitor (P5726 and P0004), all purchased form Sigma, were added per 5x10^6^ cells. Protein concentrations were determined with the aid of a colorimetric assay based on the Bradford-dye binding method (Biorad Laboratories AG, Cressier, Switzerland), according to manufacturer’s instructions.

### Immunoblots

For ZO-1 and β-catenin quantification, proteins were denatured with sodium-dodecyl sulfate (SDS) and then loaded onto a polyacrylamide gel. After separation via electrophoresis, proteins were transferred to nitrocellulose membranes (material provided by Biorad and Sigma). Membranes were then blocked in 5% non-fat dry milk in tris-buffered saline (TBS; **S1 Table**). Primary antibodies were incubated at 4°C overnight with gentle shaking. Membranes were washed with TBS and 0.1% Tween 20 (Sigma) and consecutively incubated in the presence of the corresponding horseradish peroxidase (HRP)-conjugated secondary antibody. Band detection was performed with enhanced chemiluminescent substrate and visualized using luminescent image analyzer LAS-3000 (Fujifilm, Dielsdorf, Switzerland). Blot quantification was performed using ImageJ software (ImageJ, NIH, Maryland, USA). For protein detection with Western blotting, antibodies against ZO-1 were provided by Life technologies, for histone 3 by Cell Signaling (CST, Leiden, Netherlands), while secondary (HRP-coupled) antibodies were provided by Sigma and β-catenin antibodies by BD (Biosciences, Allschwil, Switzerland). The protein amount was normalized to the mean protein expression in normoxia and adjusted for the histone 3 loading control.

### Immunofluorescence

Cells were grown on poly-D-lysine hydrobromide (50–100μg (cm^2^)^-1^ Sigma) and collagen-coated glass cover slips (Karl Hecht GmbH&Co KG, Sondheim, Germany) and exposed to normoxia or severe hypoxia prior to 4h reoxygenation with or without sevoflurane as described. The monoclonal mouse anti-β-catenin antibody was used at a dilution of 1:500 (BD), monoclonal mouse anti-ZO-1 antibody at a dilution of 1:100 and phalloidin-Alexa 568 at 1:30 (both Life Technologies). The secondary anti-mouse Alexa-Fluor 488 antibody (Life technologies) was applied at a dilution of 1:500 and 4’, 6-diamidino-2-phenylindole (DAPI, Roche Diagnostics, Mannheim, Germany) for nuclear staining was diluted to 1:1000.

### Microscopy

Bright field pictures of RBE4 monolayers were taken with an inverted widefield fluorescence microscope (Leica DMI 6000, Leica Microsystems, Heerbrugg, Switzerland) and a cooled fluorescence monochrome camera (Leica DFC 350 FX, 1392 x 1040 pixel, pixel size 6.4 μm.

### DNA quantification assay

In order to detect changes in DNA content, a quantification assay was performed using Bisbenzimide Hoechst 33342 (Sigma). Fluorescence was measured with a fluorometer (Tecan) at ex/em 360/460nm.

### LDH release assay

For quantification of lactate dehydrogenase (LDH) release, the CytoTox®Non-Radioactive Cytotoxicity Assay (Promega, Dübendorf, Switzerland) was used. With the aid of this enzymatic assay, LDH, a cytosolic enzyme is measured in cell culture supernatants at an absorbance of 492nm with a spectrometer (Tecan). For maximum lysis control, lysis buffer was added 4 hours prior to detection.

### Caspase assay

In order to quantify the cellular caspase activity, we used a selective caspase substrate for the caspases 3, 7 and 8 coupled to a fluorescent dye (Ac-Asp-Glu-Val-Asp-AMC; 3171v, Peptanova, Sandhausen, Germany). When the caspases are present, the fluorogenic AMC residue is cleaved and can be detected at ex/em 360/ 465nm with a fluorometer (Tecan).

### Flow cytometry and cell size measurement

For flow and imaging cytometry cells were washed twice with 2.5mmol EDTA in PBS, detached with trypsin-EDTA solution (0.05%) (Thermo, Reinach, Switzerland) and put through a 35μm cell strainer (Falcon, Corning, USA) after resuspension. The fixable viability dye eFluor® 660 (e Bioscience, Hatfield, UK) was used for viability analysis in accordance with the manufacturer’s instructions. For cell analysis, BD FACS Canto II (BD) and Image Stream X Mark II (Merck, Millipore, Schaffhausen, Switzerland) were used. Doublets were excluded with the aid of a pulse geometry gate (FS-H x FS-A) in the flow cytometer and microscopically in the Image Stream analysis. Dead cells were excluded upon staining. For the flow cytometry analysis, the FSC-A voltage values of the first 500 cells in each experiment were analyzed and normalized to normoxia. For image stream analysis, cell size for every measured cell was determined. ANOVA and Bonferroni correction was used for comparison of the different conditions.

### VEGF analysis using enzyme linked immunoabsorbant assays

ELISA kits for VEGF were obtained from R&D Systems Europe (Abingdon, UK). Assays were performed according to the manufacturer´s instructions. Cells were grown on collagen-coated plates at a density of 50,000 ml^-1^ for 2 days and exposed to severe hypoxia (24 hours) and reoxygenation (4 hours) with or without sevoflurane as described above. Afterwards, cell supernatants were collected and VEGF protein content quantified.

### Addition of VEGF antibody and recombinant VEGF during the reoxygenation period

In order to assess the effect of VEGF during the reoxygenation period, permeability experiments were performed as described above. During the 4-hour reoxygenation period, VEGF antibody (AF564 goat anti-rat VEGF antibody, R&D, Abingdon, UK) was added at a concentration of 1μg/ml, the corresponding goat IgG served as a control as previously described [22]. In order to assess whether the effect of sevoflurane can be reversed by recombinant VEGF (rVEGF), VEGF protein was added at a concentration of 50ng/ml [23].

### Data analysis, statistics and graphs

Data are graphed with mean values with standard deviation represented as whiskers. Each assay was performed at least three times independently. Statistical analysis was performed using either paired, two tailed-t-tests for two groups or one way-analysis of variance (ANOVA) for more than two groups, with Bonferroni’s correction for multiple comparisons. Flow cytometry data was analyzed using FACS DIVA 8.0.1 and FlowJo software. Image stream data were processed using Image Data Exploration and Analysis software (IDEAS) (Amnis, Seattle, WA, USA). Densitometry analysis and areas of phalloidin staining were assessed with the aid of image J. To identify actin tangles, a threshold of 37 was set and pixels above 37 were defined as actin positive. Below this cut-off, actin was not clearly positive. The number of actin positive pixels per image was then counted on binary images (representing total phalloidin of the *entire cells*). For subcellular analysis, the program “CellProfiler” (www.cellprofiler.org) [24] was used to identify the *nuclei* (pixel range between 35–120 pixel units) in each image as primary objects. In a next step, the cell *cytoplasm* was identified as secondary object characterized by the area surrounding the nucleus limited by continuous F-actin tangles at the cell border. Identification of the secondary objects was performed with the aid of the watershed tool. Then, the integrated F-actin staining intensity was evaluated for each cell. Data was analyzed and graphs created using Graph Pad Prism 6 (San Diego, CA, USA) and IBM^®^ SPSS statistics 22.0.0.0.

## Results

The complete data set for the manuscript can be found in supplementary online content **S2 Table**. All relevant data are within the paper and its Supporting Information files.

### Sevoflurane postconditioning improves barrier function in rat brain endothelial cells exposed to H/R

**Fig 1:**
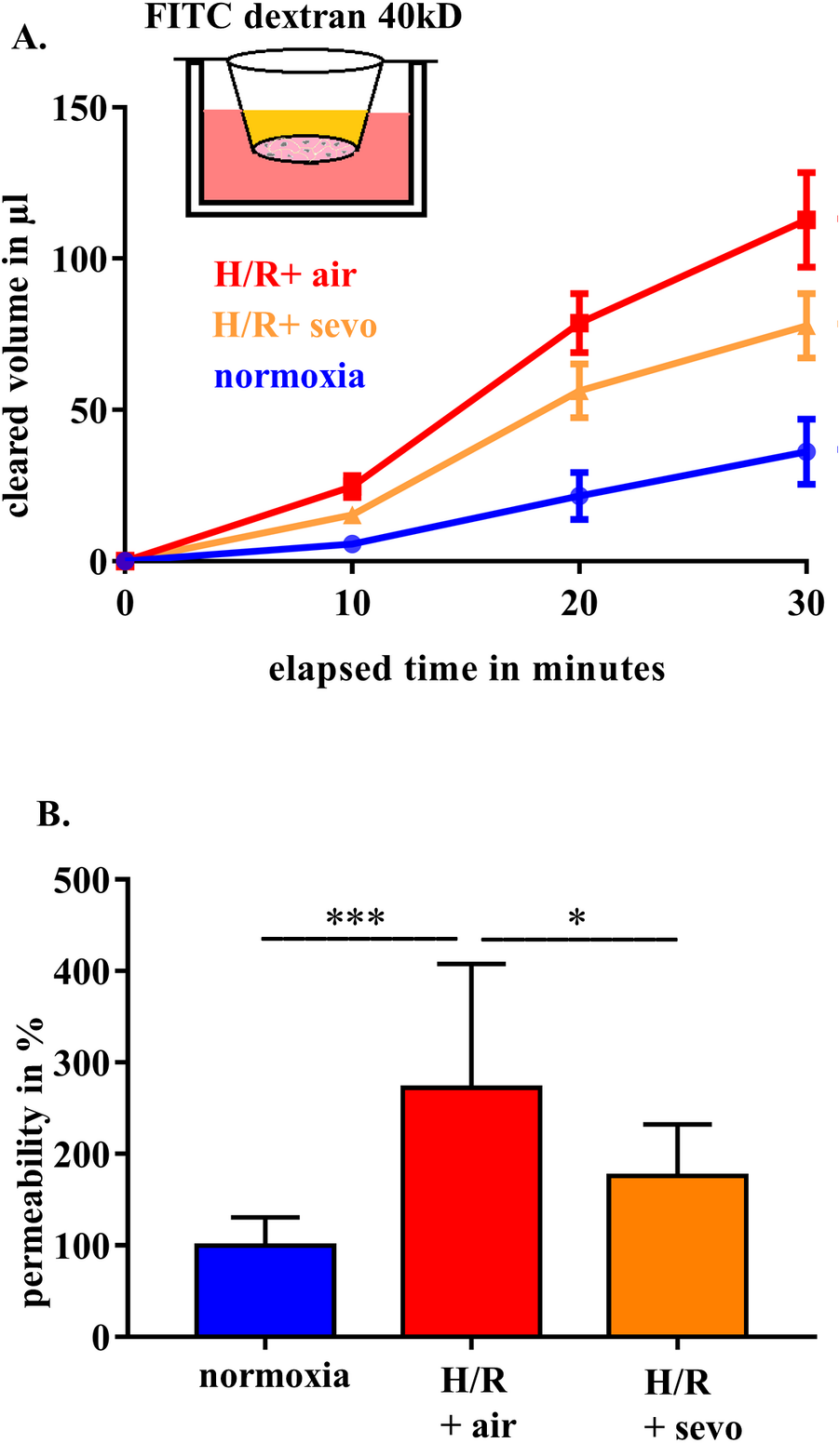
Sevoflurane and barrier function in rat brain endothelial cells in H/R injury. RBE4 cells were exposed to severe hypoxia (0.2% oxygen) for 24 hours, followed by a 4-hour period of reoxygenation in air (H/R+air) or air enriched with sevoflurane (H/R+sevo). Cells in the normoxia group remained in a normal cell culture environment with 21% oxygen for the full 28 hours. Clearance and permeability were determined using FITC-dextran. FITC clearance was measured after 10, 20 and 30 minutes (**A**). Permeability was assessed after 30 minutes and normoxia was defined as 100% (**B**). Bar graphs show mean values and standard deviations. n = 11 in each group, analyzed with ANOVA and Bonferroni correction. *** p<0.001, * p< 0.05.

When plotting the FITC-clearance against time, the curves showed a linear relationship. H/R injury induced an increase in cleared volume over time, while sevoflurane postconditioning reduced it (**Fig 1A**). The corresponding P_e_ values in normoxia were 6±1.5 x10^-3^cm/min. After 30 minutes, a 172% increase of permeability of the RBE4 monolayer was observed (normoxia: 100±31%, H/R+air: 272±135%, p<0.001). With the addition of sevoflurane during the reoxygenation period, barrier function significantly improved, leading to a 96% decrease in permeability (p<0.05; **Fig 1B**).

### Sevoflurane postconditioning preserves cellular arrangement of junction proteins in rat brain endothelial cells in H/R injury

**Fig 2:**
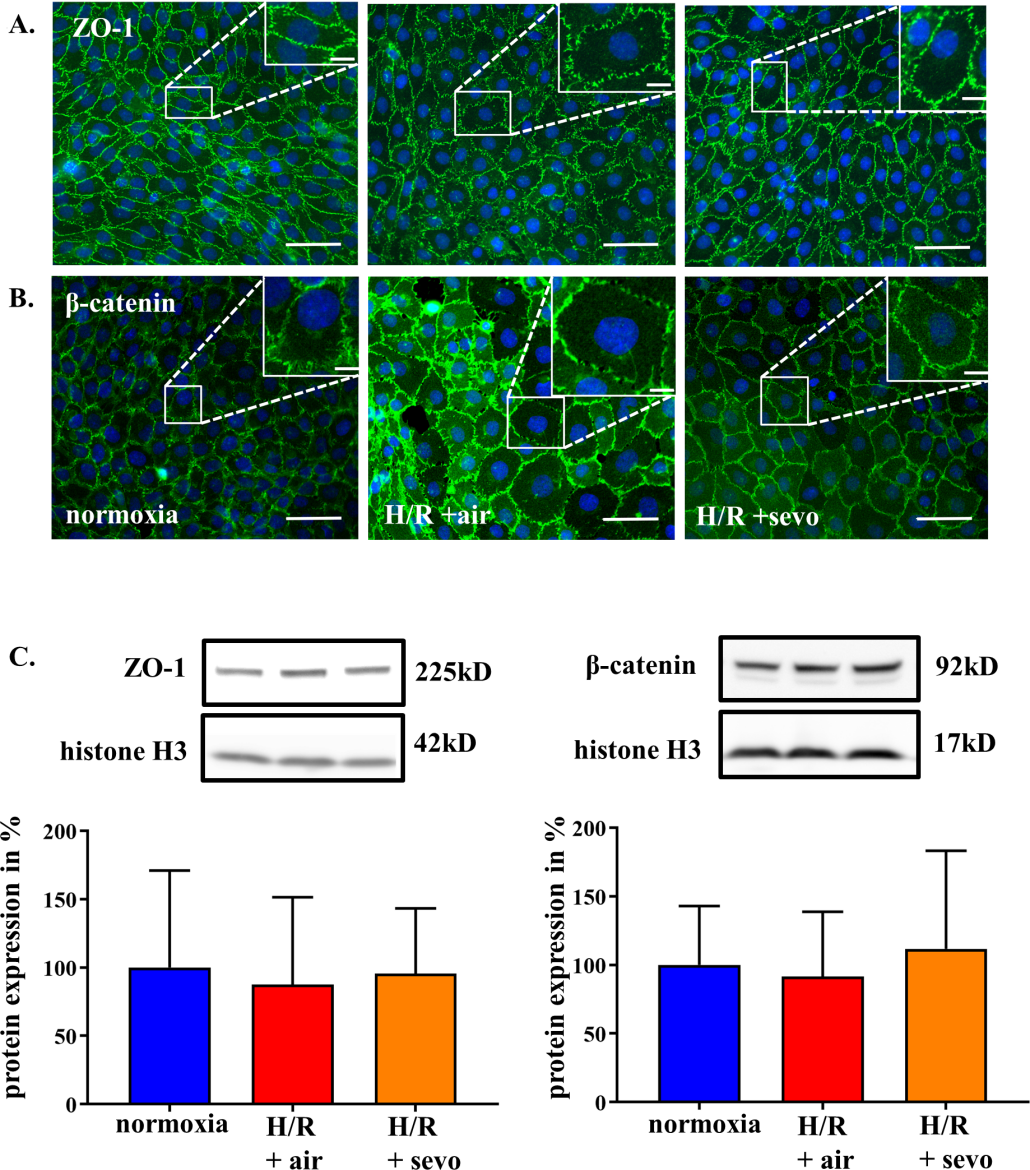
Sevoflurane and architecture of intercellular junction proteins of rat brain endothelial cells in H/R injury. RBE4 cells were exposed to severe hypoxia (0.2% oxygen) for 24 hours, followed by a 4-hour period of reoxygenation in air (H/R+air) or air enriched with sevoflurane (H/R+sevo). Cells in the normoxia group remained in a normal cell culture environment with 21% oxygen for the full 28 hours. After fixation, the junctional molecules ZO-1 (**A**) and β-catenin (**B**) were stained using according antibodies as shown in green, and the nucleus was visualized with DAPI as demonstrated in blue. Western blots were performed with according antibodies against ZO-1 and β-catenin with histone 3 as control (**C**). Bar graphs show mean values and standard deviations. n = 4 separate stainings for each group and antibody, ZO-1 and β-catenin protein analysis n = 6 per group, analyzed with ANOVA and Bonferroni correction.

Alteration of tight and adherens junction components, namely ZO-1 and β-catenin in RBE4 cells, were assessed in response to H/R+air as illustrated in **Fig 2A** and **Fig 2B**. Under H/R+air tight and adherens junction proteins in the larger cells appear disrupted with better maintained morphology in the presence of sevoflurane. For further quantification, Western blot analysis was performed and showed no alteration in the total amount of ZO-1 or β-catenin (**Fig 2C**). However, cell size increased under H/R+air, while sevoflurane partially mitigated this swelling (**Fig 2A**).

### Sevoflurane does not improve H/R-induced decrease of DNA content, increase of LDH and enhanced caspase activity

**Fig 3:**
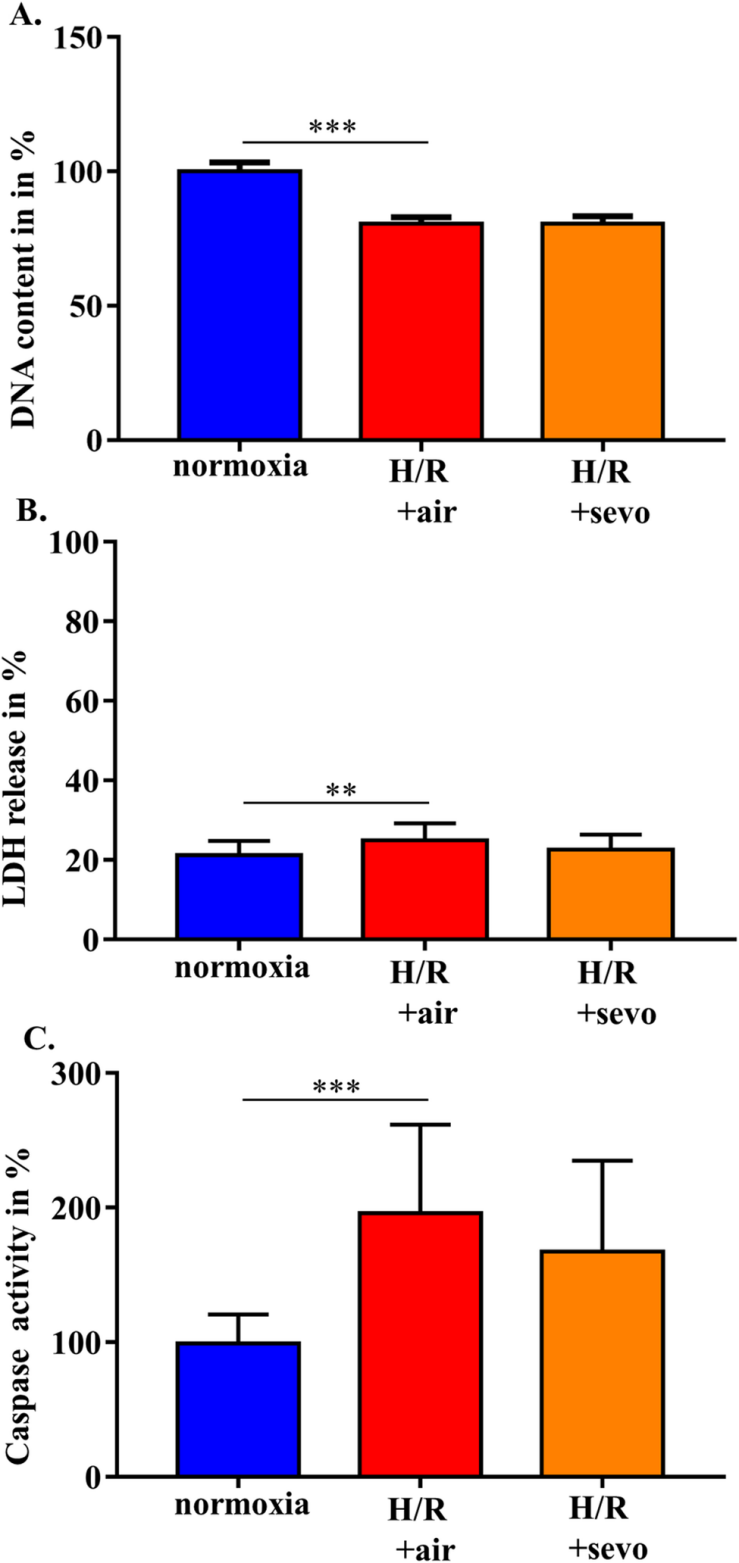
Sevoflurane and DNA content, LDH release and caspase activity in rat brain endothelial cells in H/R injury. RBE4 cells were exposed to severe hypoxia (0.2% oxygen) for 24 hours, followed by a 4-hour period of reoxygenation in air (H/R+air) or air enriched with sevoflurane (H/R+sevo). Cells in the normoxia group remained in a normal cell culture environment with 21% oxygen for the full 28 hours. Determination of DNA was performed using fluorogenic bisbenzimide (**A**). LDH release was measured with the aid of a non-radioactive cytotoxicity assay (**B**). Caspase 3/7/8 activity was assessed with a cleavage assay (**C**). Bar graphs show mean values and standard deviations. n = 18 for each group (DNA content), n = 24 for each group (LDH release and caspase activity), analyzed with ANOVA and Bonferroni correction. ** p<0.01, *** p< 0.001.

In a further step, it was determined whether changes in permeability and tight junction organization were associated with a reduced cell number and/or an increased necrosis or apoptosis rate. Compared to cells incubated under normoxic conditions, H/R decreased the DNA content by 20% (normoxia 100±3% H/R+air 81±2%, p<0.001) (**Fig 3A**), increased LDH release by 4% (normoxia 21±4%, H/R+air 25%±4%, p<0.01) (**Fig 3B**) and increased caspase activity by 71% (normoxia 100±21%, H/R+air 171±67%, p<0.001) (**Fig 3C**). However, addition of 2.2% sevoflurane during the reoxygenation period did not have an impact on these three parameters (**Fig 3**).

### Sevoflurane postconditioning preserves cell size in H/R injury

**Fig 4:**
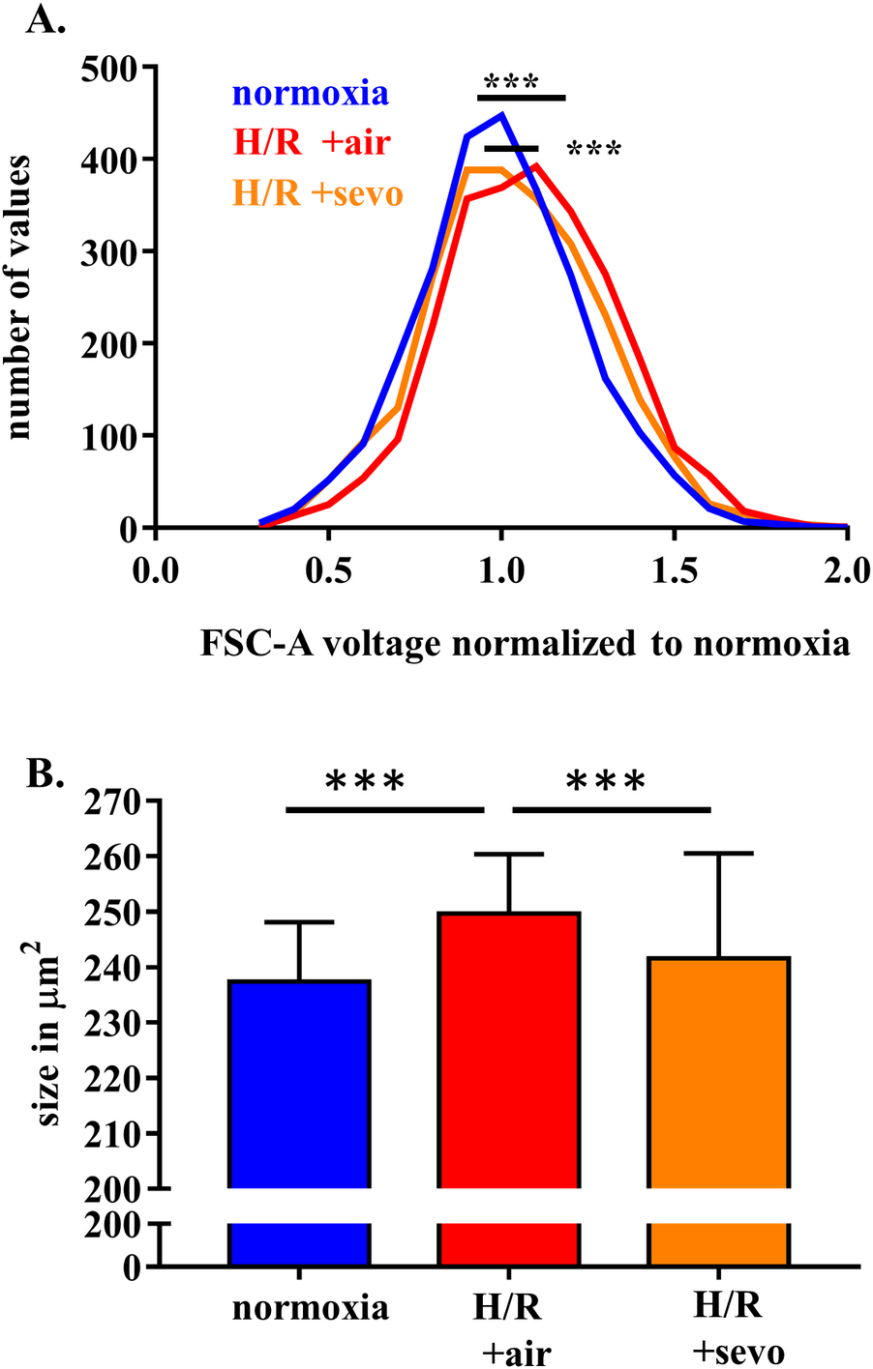
Sevoflurane and rat brain endothelial cell size in H/R injury. RBE4 cells were exposed to severe hypoxia (0.2% oxygen) for 24 hours, followed by a 4-hour period of reoxygenation in air (H/R+air) or air enriched with sevoflurane (H/R+sevo). Cells in the normoxia group remained in a normal cell culture environment with 21% oxygen for the full 28 hours. After detachment with trypsin-EDTA and viability staining, cells were analyzed with the flow cytometer or image stream, respectively. FSC-A as an indirect measure of cell size was assessed in conventional flow cytometry (**A**), while the cell size of each cell was assessed with the image stream method (**B**). The histogram shows the distribution pattern of FSC-A seen in the flow cytometry. n = 5 independent experiments, with 500 cells analyzed in each condition. The bar graph shows mean values and standard deviations. n = 4 Image stream X experiments, at least 20,000 cells analyzed in each condition. Analysis with one way ANOVA and Bonferroni correction. *** p<0.001.

To verify observed changes of cell size in **Fig 2** further experiments were performed using flow cytometry and image stream. H/R induced a mean forward scatter area (FSC-A) increase by 8.6% (p<0.001), while sevoflurane treatment decreased this change to 3.5% (p<0.001) (**Fig 4A**). This FSC-A-change correlated well with cell size changes in image stream analysis, which revealed a mean area of 237μm^2^ for cells grown in normoxia, a cell size increase after H/R, which was more pronounced when cells were reoxygenated with air (average area 250μm^2^) compared to reoxygenation with sevoflurane (average area 242μm^2^). These changes were significant for each experiment (p<0.001 for normoxia vs. H/R + air and p<0.001 for H/R + air vs. H/R + sevo) (**Fig 4B**).

### Sevoflurane acts on F-actin cytoskeleton

**Fig 5:**
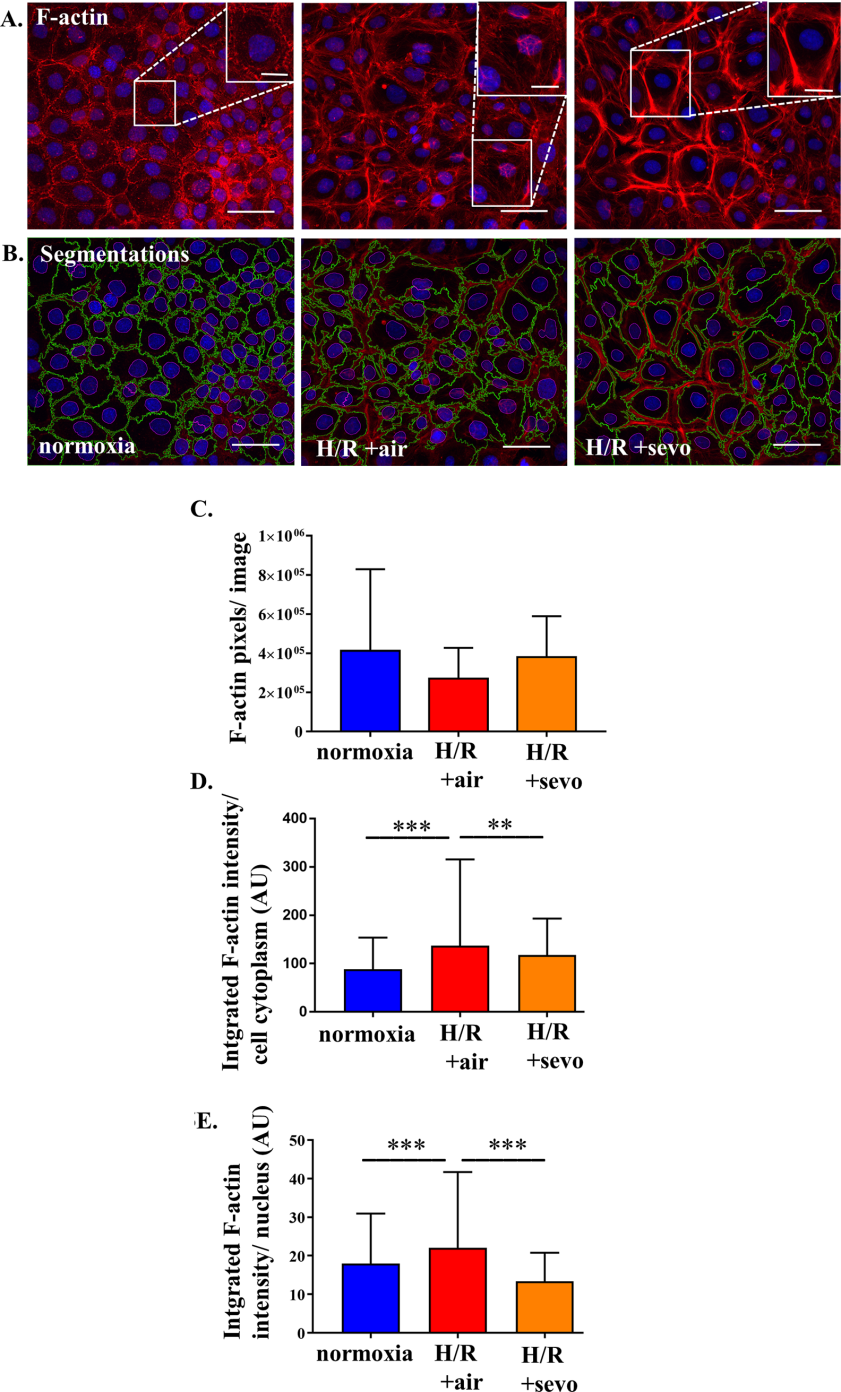
Sevoflurane and rat brain endothelial cell organization of actin cytoskeleton after H/R injury. RBE4 cells were exposed to severe hypoxia (0.2% oxygen) for 24 hours, followed by a 4-hour period of reoxygenation in air (H/R+air) or air enriched with sevoflurane (H/R+sevo). Cells in the normoxia group remained in a normal cell culture environment with 21% oxygen for the full 28 hours. Phalloidin staining visualizes F-actin tangles as seen in red, blue DAPI staining shows cell nuclei (**A**). Quantitative assessment of F-actin was performed by determination of F-actin pixels per image above the threshold of 37 in Image J (**A, C**). To further evaluate morphological changes, segmentation was performed targeting the cytoplasm surrounded with green lines and the nucleus surrounded with a cyan line (**B**). The integrated intensity in these regions of interest was then analyzed (cytoplasm, **D**) and (nucleus, **E**). n = 3 independent experiments with 3 analyzed images per condition per experiment. Analysis of the mean of each experiment with one way ANOVA and Bonferroni correction.

In order to know whether the volume changes translate into changes of the cytoskeleton phalloidin stainings were performed. RBE4 cells grown in normoxia showed predominantly membrane associated F-actin, while after H/R the normal F-actin structure was disrupted. In the presence of sevoflurane during reoxygenation F-actin was associated in bundles and redirected to the cell membrane (**Fig 5A and Fig 5B**). While this qualitative difference was observed, total F-actin did not differ (**Fig 5C**). Segmentation of each cell into cytoplasm and nucleus revealed an increase of F-actin in response to H/R+air both in the cytoplasm and in the nuclei compared to H/R+sevo (**Fig 5D** and **Fig 5E**), with F-actin primarily localized at the cell membrane. Detailed evaluation revealed that the amount of F-actin within the cell cytoplasm increased by 55% (p<0.001) when exposed to H/R+air. Reoxygenation in the presence of sevoflurane (H/R+sevo) attenuated this by 22% compared to H/R+air (p<0.005) (**Fig 5D**). Additionally, after H/R+air, more F-actin tangles were observed in the nucleus area compared to the normoxic condition (22% increase, p< 0.001). The reduction of integrated intensity in the nuclear area after sevoflurane postconditioning was 48% (p<0.001) **(Fig 5E).**

### Sevoflurane postconditioning attenuates increased VEGF levels after H/R injury

**Fig 6:**
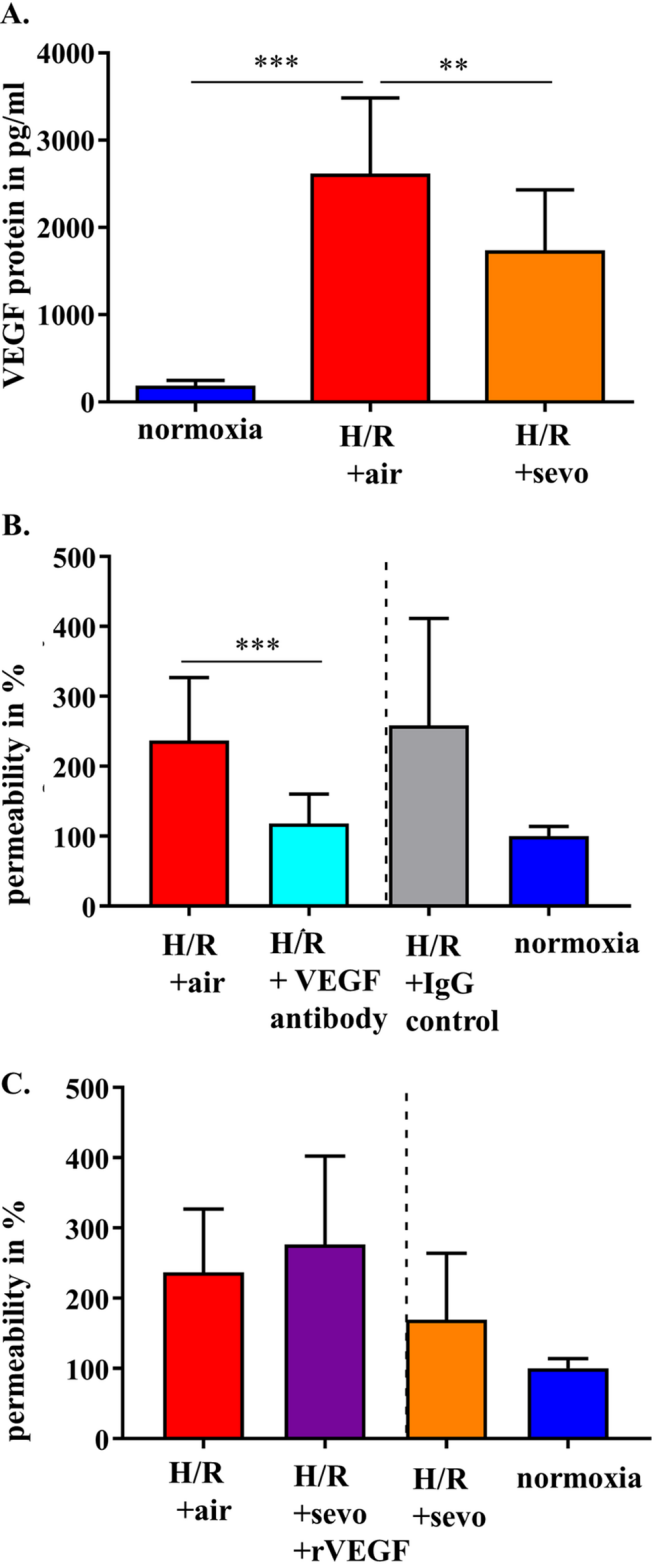
Sevoflurane and the role of VEGF expressed by rat brain endothelial cell in H/R. RBE4 cells were exposed to severe hypoxia (0.2% oxygen) for 24 hours, followed by a 4-hour reoxygenation period in air (H/R+air) or air enriched with sevoflurane (H/R+sevo). Cells in the normoxia group remained in a normal cell culture environment with 21% oxygen for the full 28 hours. VEGF protein was determined in the supernatant using ELISA (**A**). RBE4 cells were exposed to hypoxia (0.2% oxygen) for 24 hours, followed by a 4-hour reoxygenation period in air (H/R+air) or air enriched with a VEGF antibody (1μg/ml). As a control iso IgG was used. Permeability was assessed at 30 minutes using FITC-dextran. Normoxia was defined as 100% (**B**). RBE4 cells were exposed to hypoxia (0.2% oxygen) for 24 hours, followed by a 4-hour reoxygenation period in air (H/R+air) and air enriched with sevoflurane (H/R+sevo) in the presence of rVEGF (50ng/ml). Permeability was assessed at 30 minutes using FITC-dextran. Normoxia was defined as 100% (**C**). Bar graphs show mean values and standard deviations. n = 11 in normoxia and n = 15 in H/R+air and H/R+sevo (A) n = 20 in H/R+air and n = 12 in VEGF and VEGF IgG (B) n = 17 in H/R + air and n = 6 in H/R+sevo+rVEGF Analysis with one way ANOVA and Bonferroni correction. *** p<0.001 ** p<0.01.

VEGF protein concentration was measured in the RBE4 cell supernatant. This mediator VEGF was hardly detectable in normoxia at 167±82pg ml^-1^ (**Fig 6A**). However, the concentration significantly increased in response to H/R+air to 2597pg ml^-1^ (±888pg ml^-1^, p<0.001), while sevoflurane exposure reduced VEGF levels by 878pg ml^-1^ (p<0.01). In order to know whether the changes in permeability might be mediated by VEGF, this inflammatory mediator was blocked and the increase in permeability was mitigated (H/R+air 237±90% *vs*. H/R+VEGF antibody 118± 42% (p = 0.001) compared to the normoxic control (**Fig 6B**). Addition of rVEGF during reoxygenation abolished the sevoflurane-mediated beneficial effect on permeability (**Fig 6C**).

## Discussion

Blood-brain barrier disruption is highly relevant in the pathogenesis of secondary brain damage after brain injury [12, 25]. In the present study, we show for the first time that sevoflurane treatment during the early reoxygenation period following severe hypoxia promotes the maintenance of BBB structure and function in rat brain endothelial cells. The permeability patterns of the RBE4 grown on Transwells™ were comparable with the data shown by Rist et al. [21] with P_e_-values of 6x10^-3^cm per minute in normoxia.

The increased permeability for macromolecular FITC dextran after H/R reflects a severe barrier disruption in RBE4 monolayers, consistent with previously published data [26]. These changes can be explained by dysregulated tight and adherens junction proteins [27], which normally limit the paracellular flux [28] and orchestrate intercellular contact formation [29]. ZO-1-depleted cells lack functional junctions [29], and endothelial cells with conditional inactivation of β-catenin exhibit reduced cell adhesion and increased permeability [30]. Interestingly, in our experiments, the amount of tight and adherens junction proteins tested was not reduced after H/R, suggesting that application of sevoflurane helps to reorganize the existing protein structures without any quantitative change. Although beyond the scope of our experiments, it is tempting to further assess if severe hypoxia alone modifies junctional integrity and to which extend the reoxygenation process changes these dynamics.

We showed that H/R injury in brain endothelial cells induces both necrosis as reflected by decreased DNA content and increased LDH release as well as apoptosis, which was defined by increased caspase activity. One would expect an improved barrier function in the presence of sevoflurane lowering death rate. However, the application of sevoflurane did not impact on necrosis or on apoptosis. Sevoflurane rather influenced cell size and structural organization of the endothelial cells and thereby counteracted impaired barrier function.

Swelling of the endothelial cells due to edema formation is described as a prominent feature after H/R [31], and may indeed lead to disruption of the junctions. It is quantified by both flow cytometry and image stream. While the voltage of the forward scatter in flow cytometry analysis can both be modified by cell size and the refractive index differences of fluids and cells [32], microscopical analysis via image stream can accurately determine the cell size [33]. When comparing the images of cells either reoxygenated with air or with sevoflurane, computational analysis identified the cell size as differentiating factor between the treatment groups.

One of the most potent vascular permeability factors is VEGF [6]. In our experimental setting, this signaling molecule was significantly upregulated in response to H/R. At the same time, sevoflurane reduced VEGF expression relative to H/R without sevoflurane postconditioning. VEGF increases nuclear levels of β-catenin and modifies its subcellular distribution without changing the total cell amount of this adherens junction protein [34]. Besides this direct action on tight and adherens junction proteins [34, 35]. VEGF may also have an indirect effect on cell size [36]. *In vivo* it has been shown that an early blockade of VEGF reduces brain tissue edema formation in mice after stroke [37] and that it also attenuates on a cellular level the extent of cell swelling induced by cytotoxic edema after cerebral venous infarction in rats [38]. In our experiments, we could demonstrate on a cellular level, that H/R induces cell swelling, which is mitigated by sevoflurane treatment, possibly via downregulation of VEGF.

It is important to realize that F-actin structure and regulation [39], which is supposed to be involved in the spatial control and stabilization of cellular junctions [40], may be another component being influenced in the presence of sevoflurane. H/R injury caused disruption of F-actin, sevoflurane, however, stabilized arrangement of F-actin.

While an established and elegant model [19], our *in vitro* approach has its limitations. The effects detected are specific for endothelial cells and cannot reflect the full complexity of the cellular interplay at the BBB including astrocytes and pericytes. Moreover, every immortalized cell line is modified to allow cell processing and tissue specific functions may not be fully represented [41]. Finally, although we detected that sevoflurane reduces VEGF protein secretion, which is potentially involved in junctional and cellular changes, we did not elucidate the detailed signaling pathway.

In summary, we show that the impairment of barrier function in brain endothelial cells is improved by sevoflurane postconditioning. This anesthetic acts on tight and adherens junctions, possibly through the attenuation of injury-induced production of VEGF. This signaling pathway may be an interesting future drug target, but needs to be further assessed in detail in *in vivo* studies.

## Supporting information

S1 Table Composition of phosphate-buffered saline as tris-buffered saline. (DOCX)

S2 Table Relevant data of the study. All data have been summarized in this table.
(XLSX)

## References

[1] Daneman R. The blood-brain barrier in health and disease. Ann Neurol. 2012;72(5):648–72. doi: 10.1002/ana.23648 . 23280789 10.1002/ana.23648

[2] Abbott NJ, Friedman A. Overview and introduction: the blood-brain barrier in health and disease. Epilepsia. 2012;53 Suppl 6:1–6. doi: 10.1111/j.1528-1167.2012.03696.x ; PubMed Central PMCID: PMC3625728. 23134489 10.1111/j.1528-1167.2012.03696.x PMC3625728

[3] Tietz S, Engelhardt B. Brain barriers: Crosstalk between complex tight junctions and adherens junctions. J Cell Biol. 2015;209(4):493–506. doi: 10.1083/jcb.201412147 ; PubMed Central PMCID: PMCPMC4442813. 26008742 10.1083/jcb.201412147 PMC4442813

[4] Pillai DR, Dittmar MS, Baldaranov D, Heidemann RM, Henning EC, Schuierer G, et al Cerebral ischemia-reperfusion injury in rats—a 3 T MRI study on biphasic blood-brain barrier opening and the dynamics of edema formation. J Cereb Blood Flow Metab. 2009;29(11):1846–55. doi: 10.1038/jcbfm.2009.106 ; PubMed Central PMCID: PMCPMC2848453. 19654585 10.1038/jcbfm.2009.106 PMC2848453

[5] Chodobski A, Chung I, Kozniewska E, Ivanenko T, Chang W, Harrington JF, et al Early neutrophilic expression of vascular endothelial growth factor after traumatic brain injury. Neuroscience. 2003;122(4):853–67. . 14643756 10.1016/j.neuroscience.2003.08.055

[6] Senger DR, Van de Water L, Brown LF, Nagy JA, Yeo KT, Yeo TK, et al Vascular permeability factor (VPF, VEGF) in tumor biology. Cancer Metastasis Rev. 1993;12(3–4):303–24. . 8281615 10.1007/BF00665960

[7] Olsson AK, Dimberg A, Kreuger J, Claesson-Welsh L. VEGF receptor signalling—in control of vascular function. Nat Rev Mol Cell Biol. 2006;7(5):359–71. doi: 10.1038/nrm1911 . 16633338 10.1038/nrm1911

[8] Hippenstiel S, Krull M, Ikemann A, Risau W, Clauss M, Suttorp N. VEGF induces hyperpermeability by a direct action on endothelial cells. Am J Physiol. 1998;274(5 Pt 1):L678–84. . 9612282 10.1152/ajplung.1998.274.5.L678

[9] Fischer S, Wobben M, Marti HH, Renz D, Schaper W. Hypoxia-induced hyperpermeability in brain microvessel endothelial cells involves VEGF-mediated changes in the expression of zonula occludens-1. Microvasc Res. 2002;63(1):70–80. doi: 10.1006/mvre.2001.2367 . 11749074 10.1006/mvre.2001.2367

[10] Gavard J, Gutkind JS. VEGF controls endothelial-cell permeability by promoting the beta-arrestin-dependent endocytosis of VE-cadherin. Nat Cell Biol. 2006;8(11):1223–34. doi: 10.1038/ncb1486 . 17060906 10.1038/ncb1486

[11] Bae MJ, Lee YM, Kim YH, Han HS, Lee HJ. Utilizing Ultrasound to Transiently Increase Blood-Brain Barrier Permeability, Modulate of the Tight Junction Proteins, and Alter Cytoskeletal Structure. Curr Neurovasc Res. 2015;12(4):375–83. . 26238467 10.2174/1567202612666150731105831

[12] Obermeier B, Daneman R, Ransohoff RM. Development, maintenance and disruption of the blood-brain barrier. Nat Med. 2013;19(12):1584–96. doi: 10.1038/nm.3407 24309662 10.1038/nm.3407 PMC4080800

[13] De Hert SG, Van der Linden PJ, Cromheecke S, Meeus R, Nelis A, Van Reeth V, et al Cardioprotective properties of sevoflurane in patients undergoing coronary surgery with cardiopulmonary bypass are related to the modalities of its administration. Anesthesiology. 2004;101(2):299–310. . 15277911 10.1097/00000542-200408000-00009

[14] Beck-Schimmer B, Breitenstein S, Urech S, De Conno E, Wittlinger M, Puhan M, et al A randomized controlled trial on pharmacological preconditioning in liver surgery using a volatile anesthetic. Ann Surg. 2008;248(6):909–18. doi: 10.1097/SLA.0b013e31818f3dda . 19092335 10.1097/SLA.0b013e31818f3dda

[15] Voigtsberger S, Lachmann RA, Leutert AC, Schlapfer M, Booy C, Reyes L, et al Sevoflurane ameliorates gas exchange and attenuates lung damage in experimental lipopolysaccharide-induced lung injury. Anesthesiology. 2009;111(6):1238–48. doi: 10.1097/ALN.0b013e3181bdf857 . 19934867 10.1097/ALN.0b013e3181bdf857

[16] Lee HT, Ota-Setlik A, Fu Y, Nasr SH, Emala CW. Differential protective effects of volatile anesthetics against renal ischemia-reperfusion injury in vivo. Anesthesiology. 2004;101(6):1313–24. . 15564938 10.1097/00000542-200412000-00011

[17] Adamczyk S, Robin E, Simerabet M, Kipnis E, Tavernier B, Vallet B, et al Sevoflurane pre- and post-conditioning protect the brain via the mitochondrial K ATP channel. Br J Anaesth. 2010;104(2):191–200. doi: 10.1093/bja/aep365 . 20086064 10.1093/bja/aep365

[18] Thal SC, Luh C, Schaible EV, Timaru-Kast R, Hedrich J, Luhmann HJ, et al Volatile anesthetics influence blood-brain barrier integrity by modulation of tight junction protein expression in traumatic brain injury. PLoS One. 2012;7(12):e50752 doi: 10.1371/journal.pone.0050752 ; PubMed Central PMCID: PMC3519465. 23251381 10.1371/journal.pone.0050752 PMC3519465

[19] Roux F, Couraud PO. Rat brain endothelial cell lines for the study of blood-brain barrier permeability and transport functions. Cell Mol Neurobiol. 2005;25(1):41–58. . 15962508 10.1007/s10571-004-1376-9 PMC11529548

[20] Yue T, Roth Z'graggen B, Blumenthal S, Neff SB, Reyes L, Booy C, et al Postconditioning with a volatile anaesthetic in alveolar epithelial cells in vitro. Eur Respir J. 2008;31(1):118–25. doi: 10.1183/09031936.00046307 . 17898018 10.1183/09031936.00046307

[21] Rist RJ, Romero IA, Chan MW, Couraud PO, Roux F, Abbott NJ. F-actin cytoskeleton and sucrose permeability of immortalised rat brain microvascular endothelial cell monolayers: effects of cyclic AMP and astrocytic factors. Brain Res. 1997;768(1–2):10–8. . 9369295 10.1016/s0006-8993(97)00586-6

[22] Yeh WL, Lin CJ, Fu WM. Enhancement of glucose transporter expression of brain endothelial cells by vascular endothelial growth factor derived from glioma exposed to hypoxia. Mol Pharmacol. 2008;73(1):170–7. doi: 10.1124/mol.107.038851 . 17942749 10.1124/mol.107.038851

[23] Davis B, Tang J, Zhang L, Mu D, Jiang X, Biran V, et al Role of vasodilator stimulated phosphoprotein in VEGF induced blood-brain barrier permeability in endothelial cell monolayers. Int J Dev Neurosci. 2010;28(6):423–8. doi: 10.1016/j.ijdevneu.2010.06.010 ; PubMed Central PMCID: PMCPMC2918884. 20599605 10.1016/j.ijdevneu.2010.06.010 PMC2918884

[24] Carpenter AE, Jones TR, Lamprecht MR, Clarke C, Kang IH, Friman O, et al CellProfiler: image analysis software for identifying and quantifying cell phenotypes. Genome Biol. 2006;7(10):R100 doi: 10.1186/gb-2006-7-10-r100 ; PubMed Central PMCID: PMCPMC1794559. 17076895 10.1186/gb-2006-7-10-r100 PMC1794559

[25] Prakash R, Carmichael ST. Blood-brain barrier breakdown and neovascularization processes after stroke and traumatic brain injury. Curr Opin Neurol. 2015;28(6):556–64. doi: 10.1097/WCO.0000000000000248 . 26402408 10.1097/WCO.0000000000000248 PMC5267616

[26] Al Ahmad A, Gassmann M, Ogunshola OO. Maintaining blood-brain barrier integrity: pericytes perform better than astrocytes during prolonged oxygen deprivation. J Cell Physiol. 2009;218(3):612–22. doi: 10.1002/jcp.21638 . 19016245 10.1002/jcp.21638

[27] Mark KS, Davis TP. Cerebral microvascular changes in permeability and tight junctions induced by hypoxia-reoxygenation. Am J Physiol Heart Circ Physiol. 2002;282(4):H1485–94. doi: 10.1152/ajpheart.00645.2001 ; PubMed Central PMCID: PMC3918411. 11893586 10.1152/ajpheart.00645.2001 PMC3918411

[28] Komarova Y, Malik AB. Regulation of endothelial permeability via paracellular and transcellular transport pathways. Annu Rev Physiol. 2010;72:463–93. doi: 10.1146/annurev-physiol-021909-135833 . 20148685 10.1146/annurev-physiol-021909-135833

[29] Tornavaca O, Chia M, Dufton N, Almagro LO, Conway DE, Randi AM, et al ZO-1 controls endothelial adherens junctions, cell-cell tension, angiogenesis, and barrier formation. J Cell Biol. 2015;208(6):821–38. doi: 10.1083/jcb.201404140 ; PubMed Central PMCID: PMCPMC4362456. 25753039 10.1083/jcb.201404140 PMC4362456

[30] Cattelino A, Liebner S, Gallini R, Zanetti A, Balconi G, Corsi A, et al The conditional inactivation of the β-catenin gene in endothelial cells causes a defective vascular pattern and increased vascular fragility. The Journal of Cell Biology. 2003;162(6):1111–22. doi: 10.1083/jcb.200212157 12975353 10.1083/jcb.200212157 PMC2172846

[31] Liang D, Bhatta S, Gerzanich V, Simard JM. Cytotoxic edema: mechanisms of pathological cell swelling. Neurosurg Focus. 2007;22(5):E2 ; PubMed Central PMCID: PMCPMC2740913. 17613233 10.3171/foc.2007.22.5.3 PMC2740913

[32] Tzur A, Moore JK, Jorgensen P, Shapiro HM, Kirschner MW. Optimizing optical flow cytometry for cell volume-based sorting and analysis. PLoS One. 2011;6(1):e16053 doi: 10.1371/journal.pone.0016053 ; PubMed Central PMCID: PMCPMC3024321. 21283800 10.1371/journal.pone.0016053 PMC3024321

[33] Basiji DA, Ortyn WE, Liang L, Venkatachalam V, Morrissey P. Cellular image analysis and imaging by flow cytometry. Clin Lab Med. 2007;27(3):653–70, viii. doi: 10.1016/j.cll.2007.05.008 ; PubMed Central PMCID: PMCPMC2034394. 17658411 10.1016/j.cll.2007.05.008 PMC2034394

[34] Ilan N, Tucker A, Madri JA. Vascular endothelial growth factor expression, beta-catenin tyrosine phosphorylation, and endothelial proliferative behavior: a pathway for transformation? Lab Invest. 2003;83(8):1105–15. . 12920240 10.1097/01.lab.0000083531.84403.8b

[35] Wang W, Dentler WL, Borchardt RT. VEGF increases BMEC monolayer permeability by affecting occludin expression and tight junction assembly. Am J Physiol Heart Circ Physiol. 2001;280(1):H434–40. . 11123261 10.1152/ajpheart.2001.280.1.H434

[36] Sims D, Duchek P, Baum B. PDGF/VEGF signaling controls cell size in Drosophila. Genome Biol. 2009;10(2):R20 doi: 10.1186/gb-2009-10-2-r20 ; PubMed Central PMCID: PMCPMC2688285. 19216764 10.1186/gb-2009-10-2-r20 PMC2688285

[37] van Bruggen N, Thibodeaux H, Palmer JT, Lee WP, Fu L, Cairns B, et al VEGF antagonism reduces edema formation and tissue damage after ischemia/reperfusion injury in the mouse brain. J Clin Invest. 1999;104(11):1613–20. doi: 10.1172/JCI8218 ; PubMed Central PMCID: PMCPMC409867. 10587525 10.1172/JCI8218 PMC409867

[38] Kimura R, Nakase H, Tamaki R, Sakaki T. Vascular endothelial growth factor antagonist reduces brain edema formation and venous infarction. Stroke. 2005;36(6):1259–63. doi: 10.1161/01.STR.0000165925.20413.14 . 15879344 10.1161/01.STR.0000165925.20413.14

[39] Doherty GJ, McMahon HT. Mediation, modulation, and consequences of membrane-cytoskeleton interactions. Annu Rev Biophys. 2008;37:65–95. doi: 10.1146/annurev.biophys.37.032807.125912 . 18573073 10.1146/annurev.biophys.37.032807.125912

[40] Hayes S. Adherens junctions: which way is up? Nat Cell Biol. 2006;8(10):1052 doi: 10.1038/ncb1006-1052 . 17013418 10.1038/ncb1006-1052

[41] Pan C, Kumar C, Bohl S, Klingmueller U, Mann M. Comparative proteomic phenotyping of cell lines and primary cells to assess preservation of cell type-specific functions. Mol Cell Proteomics. 2009;8(3):443–50. doi: 10.1074/mcp.M800258-MCP200 ; PubMed Central PMCID: PMCPMC2649808. 18952599 10.1074/mcp.M800258-MCP200 PMC2649808

