# Supplementary material for: Sevoflurane protects rat brain endothelial barrier structure and function after hypoxia-reoxygenation injury
Source: PLoS One. 2017 Oct 12;12(10):e0184973. doi: 10.1371/journal.pone.0184973 (PMC5638245; doi:10.1371/journal.pone.0184973)
Supplement: S1 Table — (DOCX) [file pone.0184973.s001.docx]

**S1 Table: Phosphate-buffered saline and tris-buffered saline**

1. **Composition of phosphate-buffered saline (PBS 10x):**

sodiumchloride 80g; disodiumhydrogenphosphate 17g; potassiumhydrogenphosphate 4g, diluted in distilled water with target pH of 7.4

1. **Composition of tris-buffered saline (TBS 10x):**

tris base 24g; sodium chloride 88g, dissolved in 900ml distilled water and adjusted to a pH of 7.6 with HCl 9M
